# LPTK: a linguistic pattern-aware dependency tree kernel approach for the BioCreative VI CHEMPROT task

**DOI:** 10.1093/database/bay108

**Published:** 2018-10-22

**Authors:** Neha Warikoo, Yung-Chun Chang, Wen-Lian Hsu

**Affiliations:** 1 Institute of Biomedical Informatics, National Yang-Ming University, Taipei, Taiwan; 2 Bioinformatics Program, Taiwan International Graduate Program, Institute of Information Science, Academia Sinica, Taipei, Taiwan; 3 Institute of Information Science, Academia Sinica, Taipei, Taiwan; 4 Graduate Institute of Data Science, Taipei Medical University, Taipei, Taiwan

## Abstract

Identifying the interactions between chemical compounds and genes from biomedical literatures is one of the frequently discussed topics of text mining in the life science field. In this paper, we describe Linguistic Pattern-Aware Dependency Tree Kernel, a linguistic interaction pattern learning method developed for CHEMPROT task–BioCreative VI, to capture chemical–protein interaction (CPI) patterns within biomedical literatures. We also introduce a framework to integrate these linguistic patterns with smooth partial tree kernel to extract the CPIs. This new method of feature representation models aspects of linguistic probability in geometric representation, which not only optimizes the sufficiency of feature dimension for classification, but also defines features as interpretable contexts rather than long vectors of numbers. In order to test the robustness and efficiency of our system in identifying different kinds of biological interactions, we evaluated our framework on three separate data sets, i.e. CHEMPROT corpus, Chemical–Disease Relation corpus and Protein–Protein Interaction corpus. Corresponding experiment results demonstrate that our method is effective and outperforms several compared systems for each data set.

## Introduction

Increasing digitization of knowledge over the past decade has resulted in a multiverse of information pool, which can be tapped to explore various characteristic inferences from the data pool; these entity associations can be quantified and analyzed for varied purposes. The pinnacle of such text analysis and information identification hinges on `relation extraction’ between relevant entities mentioned within a sentence. To accomplish natural language understanding, it becomes paramount to address the task of identifying correct relational terms and entities bound in such associations. Therefore, research on relation extraction has garnered tremendous attention for many years. Extraction of these associations is even more important to life scientists as it provides them greater insight into the biological nature of the context while investigating the biological entities of interest.

Compared to yesteryears, the scale of such research has been bolstered by the introduction of precision medicine where identification of specific interaction with a range of different genotypes holds the key to treating patients effectively. Increasing granularity brought in by specific interaction identification using natural language processing can aid medical health professionals to deliver a speedy diagnosis and a targeted treatment plan to patients. In an effort to mobilize this trend, BioCreative has introduced many entity-based relation extraction tasks in recent years, including the identification of protein–protein interaction (PPI) (19), chemical-induced disease identification (CID) (15) and chemical–protein interaction (CPI) (26). We participated in BioCreative VI–CPI task and developed a Linguistic Pattern-Aware Dependency Tree Kernel (LPTK) model for studying bio-entity association types mentioned between chemicals and proteins. CPI task is a text mining-based task, where PubMed abstracts are studied to identify nature of different interaction types triggered by chemical compounds/drugs interacting with genes/proteins. These interactions refer to all direct as well as indirect regulatory associations between a chemical and protein/gene, where the association results in physical, functional or quantitative change in the gene product (protein) (26). The task guidelines specify the identification of five chemical–protein relation (CPR) classes from a set of abstracts. These classes are defined as Up Regulators—CPR3, Down Regulators—CPR4, Agonist—CPR5, Antagonist—CPR6 and Substrate/Product Of—CPR9. Apart from these, the guideline also establishes CPR10–NOT category, which is used to annotate non-interacting but co-mentioned chemical–protein entity pairs (26). CPI is an integral part of our metabolism with several different kinds of interactions regulating various biological pathways for normal functioning. Therefore, the task of extracting CPIs has potential implications in automating and upgrading the way precision medicine is conducted.

Machine learning-based methods are vastly data dependent, not only with respect to the size and structure of training data but also on the representation of data features prior to learning. These representations are responsible for identifying explanatory factors of variation within the data, which makes classifier-based learning effective (45). Representation models, be it *n*-gram word models or vectorized representations, all use statistical conditional probabilities to study sequenced associations among words for feature representation. In this study, we introduce an improved tree representation by characterizing contextual relational segment within a sentence as representative features corresponding to their invariant properties. We refer to these corpora wide-identified invariant features as `linguistic patterns’, which can communicate the essential contextual information while retaining their brevity. These invariance-based linguistic patterns are human-readable and reusable and can therefore be deployed in contextually similar relation extraction tasks. In addition, the context optimization delivered by these patterns helps reduce the data noise in tree representations, thereby improving the classification performance over other currently available lexical tree kernel representations. As a part of our relation identification strategy, we first perform a CPI pair identification (binary classification) using patterns based on non-interacting [CPR 10] versus interacting entity pairs [CPR 3, CPR 4, CPR 5, CPR 6 and CPR 9]. In the second phase, One-Vs-All (OvA, multi-class classification) approach is deployed to screen for specific CPR pair from each positively identified instance obtained during binary classification.

Our linguistic pattern representation model is generic and can be adapted for any binary relation detection task. Consequently, as an extension and corroboration of the findings from CPI task study, we extended the performance testing of our LPTK approach on two other substantial bio-entity relation identification corpora, i.e. Chemical–Disease Relation (CDR) and PPI. The model fared well with both the corpora while displaying consistency and efficiency of LPTK approach, particularly with bio-entity-based relation extraction tasks.

The remainder of this paper is organized as follows. In the next section, we review Related works documented previously. We describe details of the proposed system in the Methods section. The Experiments section shows the experiment results and presents further comparison of our work with other systems. Finally, we conclude our work in the Concluding remarks section.

## Related works

Entity-based interaction identification tasks have been introduced as a part of many challenges and collaborative endeavors in recent years. Although the CPI task provides a new compilation of CPI resources, the task of relation extraction between various biological entities isn’t a novel one. Works using dependency parser-based features for graph kernels (GKs) to sort complex PPIs were introduced by Ariola *et al*. (1) and Qian *et al*. (35). The shortest path tree built on premise of such methods impressively captured segments of text depicting true associations. In addition to dependency parser-based features, experiments have been done with normalization and pattern generation where Chang *et al*. (2) generated normalized patterns, which signified trigger words involved within the protein interactions. Screened for the pattern-associated instances, the features derived from the shortest dependency path were used to determine true protein interactions. PPI focuses on intra-sentence relations (relational associations between entities discussed within the boundaries of same sentence) as opposed to the addition of inter-sentence relation (relational associations between entities discussed across the boundaries of sentences) introduced in the CID task.

The CDR corpus in CID task–BioCreative V was developed to capture interactions between chemicals and diseases at both intra- and inter-sentence level. This added level of complexity led to changed strategy for processing the abstracts. Many learning techniques developed for CID focused on splitting learning methodologies between intra- and inter-sentence scenarios. Co-occurrence (CO) has been used to resolve ambiguity in locating heterogeneous entity pairs, upon which separate models tend to learn associations in either data sets. Xu *et al*. (44) and Li *et al*. (22) focused on exploiting sentence level and document-level classifiers to establish entity pairs involved in either structure, while Gu *et al*. (13) relied on isolating intra- and inter-sentence relations and then feeding them separately into Convolution Neural Network (CNN) and Maximum Entropy (ME) models, respectively. Coupled with learning methods, many systems incorporated features either directly based on or perfected via the use of external knowledge base (KB). Pons *et al*. (34) used Euretos Knowledge Platform (https://www.euretos-brain.com), a graph database to generate one set of features. The database provides a directed graphical structure for entity pairs with differing weights as provenance accounts for every pair across structured databases. One of the top performing systems in this task developed by Xu *et al*. (44) experimented with a set of features contingent upon external KB using MeSH, MEDI (50), SIDER (20) and CTD (8) databases for prior mentions of entity pairs in association. These lookups of external KBs aided in assigning a weight bias to identified pairs for efficient screening of association. Other than KBs, various linguistic features ranging from Bag of Words (BOW), *n*-grams word frames, term normalization, positional verb lemmas, dependency parser-based word segments and word embedding are employed in the CID task (12, 13). With this task, a gradual shift in the use of multi-kernel classifiers was noticed as depicted in the works of Gu *et al*. (12, 13), which began with the selection of deep learning techniques like CNN and Long Short-Term Memory (LSTM) for sentence-level classification in bio-entity relation identification tasks.

To date, relation extraction tasks have focused heavily on either protein or chemical interactions individually. In a pioneering effort while participating in the protein localization relation identification task, Kumlien and Craven (5) worked in part on drug–protein identification employing BOW, relational dependencies and external KB-based entities as features for Naive Bayes classifier. Later, a comprehensive NLP tool called EDGAR was developed by Rindflesch *et al*. (36), which catered for identification of drug–gene interactions specifically described in cancer-based literatures. The tool employed constructs from parsed tree in learning interactive associations. The advent of the CPI task in BioCreative VI set up a new target in recognizing chemical-based protein interactions from diverse abstracts (18). Owing to the complexity in the nature of relation description within sentences, comprehensive feature sets such as lexical, statistical and embedded representations were used in ensemble kernel setting. For instance, Peng *et al*. (33) followed an ensemble approach exploiting Support Vector Machine (SVM), CNN and Recurrent Neural Networks (RNNs) within the same model. The system used BOW, positional indices and shortest route dependency parsers as features within the classifiers. Results were obtained based on majority voting from the predictions of each classifier. Devising a layered structure for classification, Lung *et al*. (21) divided the task as binary and multi-class classification with each layer using entity pairs and triplets as features. For multi-class classification, they use additional classifiers including Logistic Regression, Linear Discriminant Analysis and Naive Bayes to assign each pair to its respective class.

Differing from most methods in the CPI task–BioCreative VI, we developed an invariant pattern consolidation method for CPIs, which is able to learn linguistic patterns from biomedical literatures. We fuse this linguistic pattern-based CPI information into dependency tree structure to capture the sophisticated nature of CPIs. This concept allows the LPTK to discriminate between various kinds of relation-associated entities in literature.

## Methods

**Figure 1:**
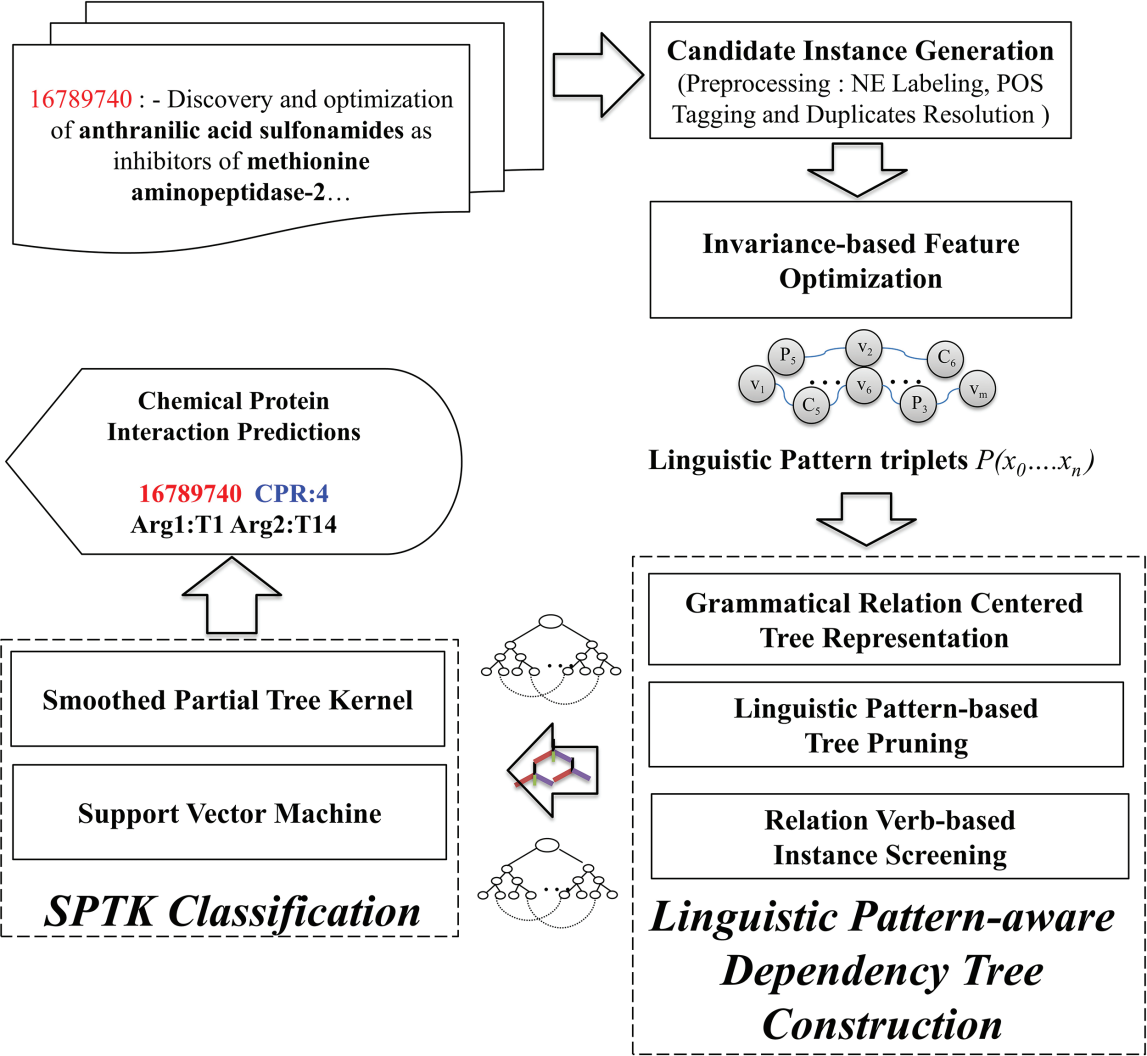
Overview of the CPI detection method.

The CHEMPROT corpus enlists abstracts from various biological literatures, supplemented with pre-annotated heterogeneous entity characterizations (i.e. chemical compounds/drugs and genes/proteins). In light of this, we present LPTK, which automatically extracts CPI entity pairs from biomedical literatures. Figure 1 illustrates the system architecture of LPTK, which encompasses three key components: `candidate instance generation, invariance-based feature optimization and linguistic pattern-aware dependency tree construction’. Each of the components mentioned maneuvers the entity-pair selection to reproduce feature characteristics of the corresponding CPR type for learning representation. The candidate instance generation first decomposes the raw abstracts into a set of candidate instances, each of which highlights a chemical and protein pair representing potential interaction. Subsequently, these entity pair and relation-word-based-tagged context frames are utilized to capture invariant interaction context using Algebraic Invariance-based scoring. Based on the grouped synonymous inferences obtained by the scores, these tagged patterns are identified as `linguistic patterns’. Candidate instances are screened against these patterns to refine the original sentence to the relevant contextual segments represented by the pattern. Corresponding quasi-pruned trees are used as representation feature for the Smooth Partial Tree Kernel (SPTK)-based classifier (16, 28, 31). Each of these stages is elucidated in detail in the following sections.

### Candidate instance generation

Candidate instance generation entails identifying sentences referred hereafter as `candidate instances’ from the original abstracts that highlight the corresponding relation verb and an entity pair representing a potential interaction. Prior to the instance generation, the data set is given a generic text preprocessing with sentence detection, entity class normalization and part-of-speech (POS) tagging. Sentence detection tools are used to segregate the sentence boundaries. Each sentence is then screened for heterogeneous entities and normalized with respective tags `Chemical’ or `GenePro’ appended with relation ID and type [e.g. ChemicalR1T3—Chemical associated with CPR3 interaction (T3) and Relation Id: 1 (R1) for the designated abstract]. The entity class-normalization module furnishes respective preprocessed sentences. Lastly, a parallel set of PoS-Tag labels corresponding to the sentences from the data set is generated using the GENIA tagger (42, 43). GENIA is preferred over the other popular PoS taggers as it is built on biological corpus and stems each word to its morphological root prior to tagging, which is quite handy while recognizing key interaction terms like `induced’ or `regulated’ as verbs, opposed to the adjective forms by other taggers.

**Table 1:** Multi-class versus same class intra-sentence distribution within CHEMPROT corpus

**Data set**	**Multi-class intra-sentence relations**		**Same class intra-sentence relations**	
	**#Relation pairs (%)**	**#Abstracts (%)**	**#Relation pairs (%)**	**#Abstracts (%)**
Training	24.91	33.76	75.08	66.23
Development	25.99	32.73	74.00	67.26
Test	31.07	41.29	68.92	58.70
Average	**27.33**	**35.92**	72.66	64.07

**Figure 2:**
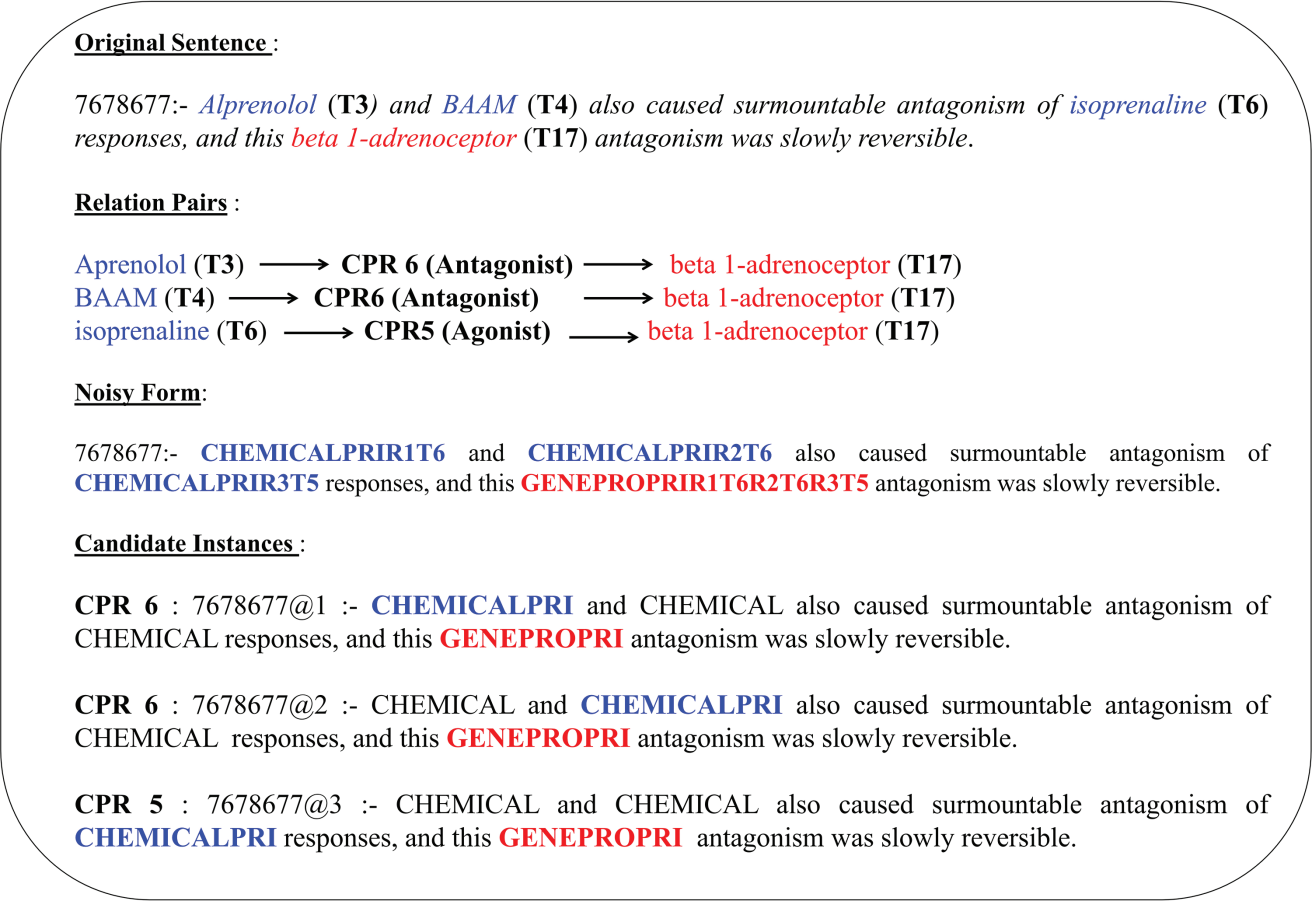
Candidate instance level entity normalization.

The abstracts in the CHEMPROT corpus often have multiple COs of heterogeneous entity pairs within a sentence. These overlapping entity references belonging to separate interaction pair mentions would contribute to additional noise in linguistic pattern detection if all relation types were to be identified from a single representation. Such multi-relation normalized tag representation would prevent identification and consolidation of contextual patterns into a generic invariant representation (owing to differently annotated entity tags). Therefore, candidate instances based on combinations of unique heterogeneous entity pair are generated from each sentence; that is, each instance is a derivative copy of the original sentence with relation mention normalized heterogeneous entity-pair combination highlighted to represent a single interaction case at a time, while the rest entity mentions are normalized to a respective generic symbol. Distribution of such multi-class versus same class intra-sentence relation is given in Table 1, highlighting that an average of 27% relation pairs in CHEMPROT corpus exist as multi-class relation co-occurring in a sentence. The same distribution also highlights that ∼35% of the abstracts are covered by multi-class intra-sentence relation scenario. An example of such a multi-class intra-sentence relation can be shown with sentence `Alprenolol **(T3)** and BAAM **(T4)** also caused surmountable antagonism of isoprenaline **(T6)** responses, and this beta 1-adrenoceptor **(T17)** antagonism was slowly reversible.’ Here we can see two separate relation types—agonist (CPR:5) and antagonists (CPR:6)—are co-located. As shown in Figure 2, the sentence has three separate relation mentions (two same class and one same distinct type) spanning over four entities (i.e. three chemicals and one gene/protein). Corresponding relation association is also shown in the figure. To resolve the sentence into a generic form, the normalized entity pairs are spread over a set of three candidate instances to formulate all potential associations per heterogeneous entity type. In addition to the primary entity pair, we also identify and normalize a `proximal verb’, i.e. the main verb nearest to the current entity-pair combination, with annotation `Relation#’, where `#’ stands for the CPR type of the entity pair. The candidate instance generation component is then able to automatically pick the proximal verb based on a comparative assessment of verb-entity distances and verb form. Linguistically, there is predominance in describing association relations between respective subject–object pairs within a smaller sentential frame using non-base/inflection (http://www.ucl.ac.uk/internet-grammar/verbs/base.htm) form verbs. Relation term normalization isn’t performed for non-interacting entity pairs, i.e. CPR 10. The abstract text candidate instance label normalization is mirrored in their corresponding PoS-tagged counterparts. PoS tag-labeled sentences are more generic in their representation, and therefore ideal in depicting the skeletal similarity of relations in contrast to the actual text. Key relation entity pairs for the PoS-tagged candidate instances are also relabeled according to their respective sentence normalized forms.

As shown in Table 2, the training data has disproportionate size of CPI relation pairs represented by CPR 3, CPR 4, CPR 5, CPR 6 and CPR 9 [referred to as positive (+ve) cases in our framework]. CPR 5 and CPR 6 have far fewer relation instance mentions than other interaction relation pairs. Their proportional imbalance (especially within training data set) leads to fewer candidate instances representations, culminating into fewer invariant linguistic patterns generated for their representation in subsequent phases. In addition, the non-interacting chemical–protein pairs identified as CPR 10 [referred to as negative (−ve) case in LPTK framework] also have low contribution size averaging to only 6.41%. CPR 10 pairs form the negative instances in our preliminary binary classification study for CPI detection. Therefore, to mitigate a lower CPR 10 linguistic feature representation due to skewed proportion of positive and negative instances, we employ cross-group combinations to identify additional negative instances within chemical–protein pairs that were present in the abstract but not mentioned in the pre-identified relation set. For the test data set, each entity-pair instance was replicated for each of the CPR types.

**Table 2:** Relation class imbalance within CHEMPROT relation data set

**Relation class**	**Training (%)**	**Development**	**Test**	**Average**
CPR 3	17.61	21.22	17.85	18.89
CPR 4	51.22	42.4	44.63	46.08
CPR 5	**3.92**	**4.45**	**5.30**	**4.55**
CPR 6	**5.32**	**7.65**	**7.84**	**6.93**
CPR 9	16.47	17.57	17.24	17.09
CPR 10	**5.43**	**6.68**	**7.12**	**6.41**

### Invariance-based feature optimization

In general, most prediction systems commonly use features based on BOW, positional distance between entities, graph routes, etc. However, these features are unable to directly highlight inferential differences among sentences with a representational overview. In this study, we attempt to explore a novel linguistic pattern representation, which can capture the summary of associative expressions from a sentence and differentiate among various classes of associations (48). Our idea is to harness the features/sections from text that are immutable upon transformation. This begs the question of what qualifies as transformation with respect to text and what kind of properties within the text can be measured as immutable corresponding to transformations. To begin with, we alter our perception of viewing transformation as dissimilarity among PoS-Tag-based frames, where all candidate instances are hypothesized to be dissimilar to each other at the initial stage; making one sentence by inference a transformed state of another. Represented in (1), this holds true in one to many association for each candidate instance.

(1) \begin{equation*} \forall\, {\vec{s}}_i,{\vec{s}}_j\in {S}_{CD} \mid {\vec{s}}_i\ne {\vec{s}}_j, \end{equation*}

where ${\vec{s}}_i$ and ${\vec{s}}_j$ are different candidate instances from ${S}_{CD}$ (set of all PoS-Tag candidate instances).

Our approach for determining immutability is heavily drawn on the concept of Algebraic Invariance showing that two separate sentences are similar in their inferential meaning if their invariant function score does not vary. Extrapolating this concept, we define immutable features using PoS-Tag frames, which appear representationally consistent across data and are helpful to describe similarity across instances. The immutability is gauged by invariant function.

The method of invariants dates back to the works of Boole and Cayley with significant contributions introduced by David Hilbert (14). The theory identifies `invariants’ as quantities, which continue to maintain their consistency even upon the introduction of relative transformational changes within the system (10). The work introduced in this paper is an attempt to apply the same concept to the biomedical text-mining domain and to explore alternative and effective ways to learn exhaustive automated patterns via an algebraic/geometric interpretation. Algebraic Invariance can be described using functionals of an *n*-degree polynomial. Invariant functional can be represented as follows:

(2) \begin{equation*} I\left({q}_{n0}.\dots {q}_{0n}\right)\equiv {\Delta}^W\times I\left({p}_{n0}.\dots {p}_{0n}\right), \end{equation*}

where *I(q)* and *I(p)* indicate the invariant function with *q_ij_* and *p_ij_* being the coefficients of *n*-degree representative polynomials *Q(x_0_..x_n_)* and *P(x_0_..x_n_),* respectively. Δ is the determinant of the representational polynomial undergone transformation, and *W* is the invariant weight.

**Figure 3:**
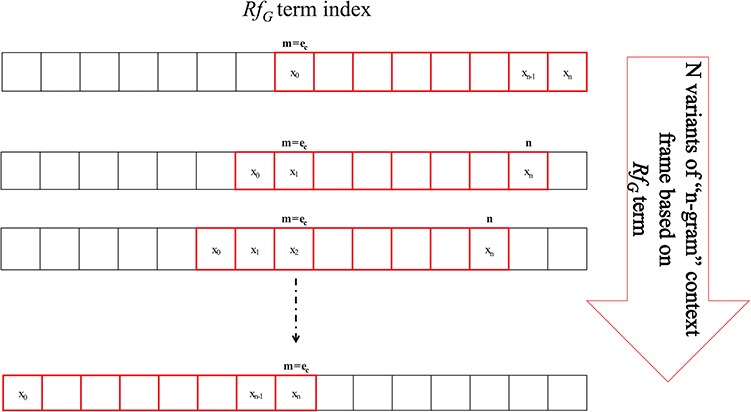
*N*-gram window-based `context-frame’ extraction.

**Figure 4:**
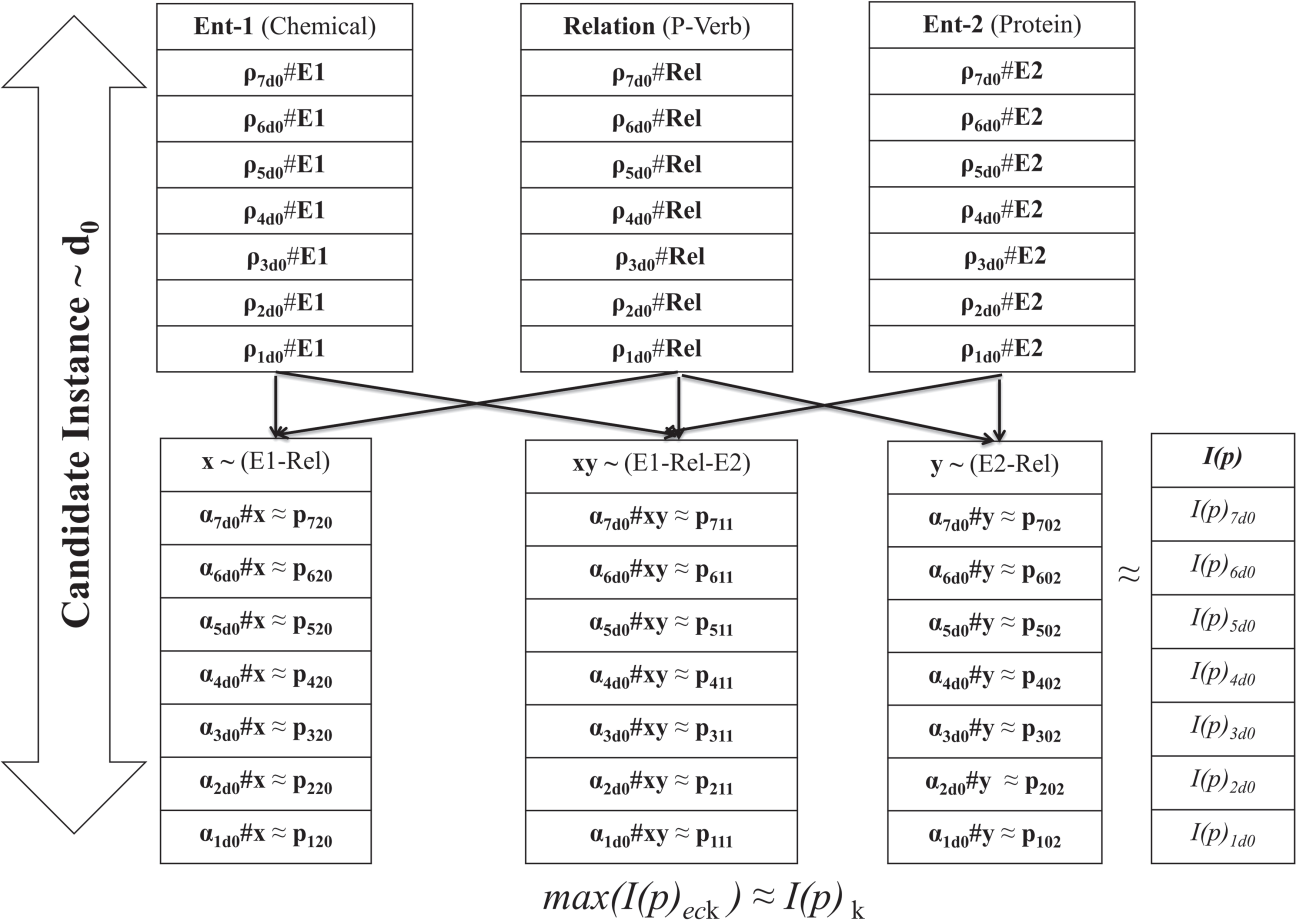
Schema for coefficient consolidation.

Any object/element can be represented in the Cartesian system (9) using a polynomial function. For the sake of this study, we consider a second order polynomial $P\left(x,y\right)=\sum {p}_{ij}{x}^i{y}^j$. Upon transformation *T*, the same polynomial $P\left(x,y\right)$ is represented by another polynomial $\left(u,v\right)=\sum {q}_{ij}{u}^i{v}^j$, bound in relation $\left(u,v\right)=T\left(x,y\right)$with original form. To employ this concept in sentient learning, we restructure our candidate instance as geometric representation (conic-sections) in Cartesian space, where quantities associated with its geometric form provide immutable features describing characteristics inherent to its sentient class. We assume a homogenous polynomial to represent geometric form of a candidate instance as shown below in (3), where each variable defines features related to the `entity-verb’ mention. We pivot our feature selection around three key referential-groups (*Rf_G_*) *viz.* `Entity1’ (chemical), `Relation’ (proximal verb) and `Entity2’ (protein/gene) to identify CPRs.

(3) \begin{equation*} P\left(x,y\right)={p}_{20}{x}^2+{p}_{11}{x}^1{y}^1+{p}_{02}{y}^2, \end{equation*}

where variables *x* and *y* describe occurrence of paired referential group `Entity1∨Relation’ and `Entity2∨Relation’, representing 2D feature space, respectively. Paired referential groups are referenced here in `OR [*∨*]’ association, i.e. if either of reference group from the pair is not mentioned in the sentence, *x* and *y* still tantamount to 1 (as `Entity 1’ and `Entity 2’ are always mentioned in each of the candidate instance). *p*_20_, *p*_02_ and *p*_11_ are coefficients of the representative polynomial, each of which represents the score given to the linguistic context frame associated with referential groups in *x* and *y*, respectively. The score is evaluated using PoS-Tag word frame-based joint probabilities for each referential group in the variable. Coefficient values vary based on the number of representative entities and context of each instance. For example, in instances with no annotated `Relation’ tag (i.e. CPR 10 or –ve instances), only `Entity 1’ and `Entity 2’ would contribute to coefficient values, impacting the polynomial conic representation for CPR 10-type instances.

To discover immutable features pertaining to conic forms based on our premise, we assume rotation (*ф* = 0) as the conformational transformation corresponding to which invariance can be judged, with minimal rotation between two conic forms signifying similarity. Using (3) and the transformation matrix, we obtain below invariant function (17):

(4) \begin{equation*} I\left({q}_{n0}\dots {q}_{0n}\right)\equiv I\left({p}_{n0}\dots {p}_{0n}\right)=\left[{p}_{20}^2+\left(\frac{p_{11}^2}{2}\right)+{p}_{02}^2\right], \end{equation*}

where *I(q)* and *I(p)* are the invariant functions for the transformed instance polynomial *Q(u,v)* and the original instance polynomial *P(x,y),* respectively. *p*_20_, *p*_11_, and *p*_02_ are the coefficients of the original polynomial function *P(x,y)*.

Each of the PoS-Tag candidate instances is screened for the three/two key *Rf_G_* terms (based on +*ve*/−*ve* entity pairs) *viz.* normalized chemical, protein and proximal verb forms. By using the PoS-Tag instance, *n*-gram word frames termed as `context frame’ are selected starting at the current *Rf_G_* term index (*e_c_*), representing shifting window frame values between 0 and *n*. Beginning with *e_c_* = 0, the iteration index terminates at *n*, which is the maximum size of the context frame as shown in Figure 3. Each of the *n*-gram context frames is evaluated for significance using (5), which is a modified representation of joint conditional probability. The modified formula takes into account all variant sub-frames succeeded and preceded by the term index *e_c_* = *m* given by [0−*m* and *n*−*m*]. This metric is able to score each sub-frame differently according to their information content and non-redundant representation within the bounds of the selected context, further optimizing the significance of context. The score is a statistical weight summary of the given context frame over other contexts in the training data for given *Rf_G_*.

(5) \begin{equation*} {\rho}_{e_ck}\kern0.75em =\left\{\begin{array}{l}\left(\frac{\sum P\left({x}_0\dots {x}_m\right)}{\sum P\left({x}_0\dots {x}_{m-1}\right)}\right)\times \left(\frac{\sum P\left({x}_0\dots {x}_n\right)}{\sum P\left({x}_0\dots {x}_m\right)}\right)\\ {}\forall \left(m={e}_c\right)\cap \left(0<m\le n\right)\cap \left(n\sim 7\right)\ \\ {}\left(\frac{\sum P\left({x}_0\dots {x}_n\right)}{\sum P\left({x}_m\dots {x}_n\right)}\right)+{\delta}_{v\approx }0.00000001000\\ {}\forall \left(m={e}_c\right)\cap \left({x}_m={x}_0\right)\cap \left(n\sim 7\right)\end{array}\right\}, \end{equation*}

where *e_c_* is the index of the *Rf_G_* term in the selected frame represented as subscript *m* sub-frames, *n* indicates the total frame size and *ρ_eck_* is the score of the context sequence for *Rf_G_* term iteration index *(e_c_)* and candidate instance *k*. *∑P(x_0_.... x_m_)* represents the number of times the respective PoS-Tag frame has occurred across all of the contexts generated from all instances. *δ_v_* is the fringe value used if a pattern is conditioned on itself. It is introduced to avoid attributing excess weight bias to a redundant pattern, which contributes low on information. Equation (5) is used to generate probability scores for all *n* × 3-context sequences for each instance.

As shown in Figure 4, for every *Rf_G_* term (e.g. chemical, protein and relation), joint probability cumulates the scores *ρ_eck_* for each of the *n*-variant context sequences. Afterwards, the paired variable-based joint probability score (*α_eck_*) corresponding to each *Rf_G_* term is determined by clubbing respective *ρ_eck_* scores. Corresponding to each of these paired variables, polynomial form of the candidate instance is generated using respective *α_eck_*, as the coefficient *p_ij_* defining constitutive characteristics of the polynomial. Consequently we obtain *n* invariant function scores *I(p)*_*ec*k_ using (4), for each iteration index *e_c_* corresponding to candidate instance Id *k*. *I(p)*_k_ representation with the highest score is identified as the characteristic geometric form. *n* is one of the hyper-parameters in our study, which was adjusted from 3 to 7 based on training results.

**Figure 5:**
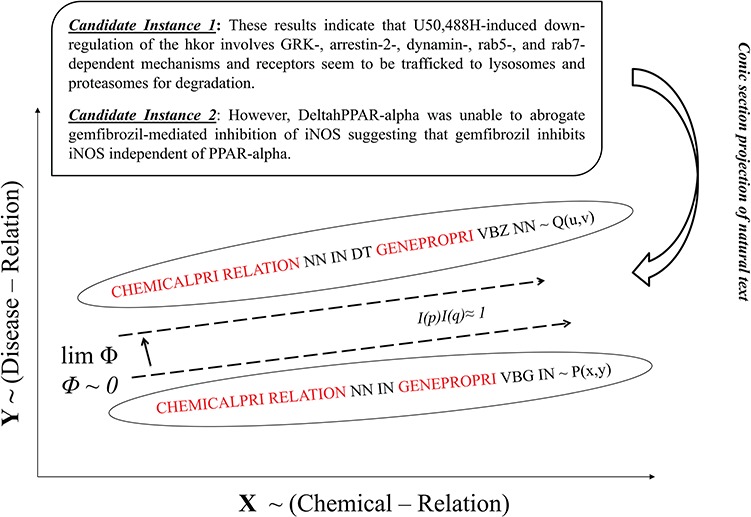
Polynomial-based spatial projection of text.

**Figure 6:**
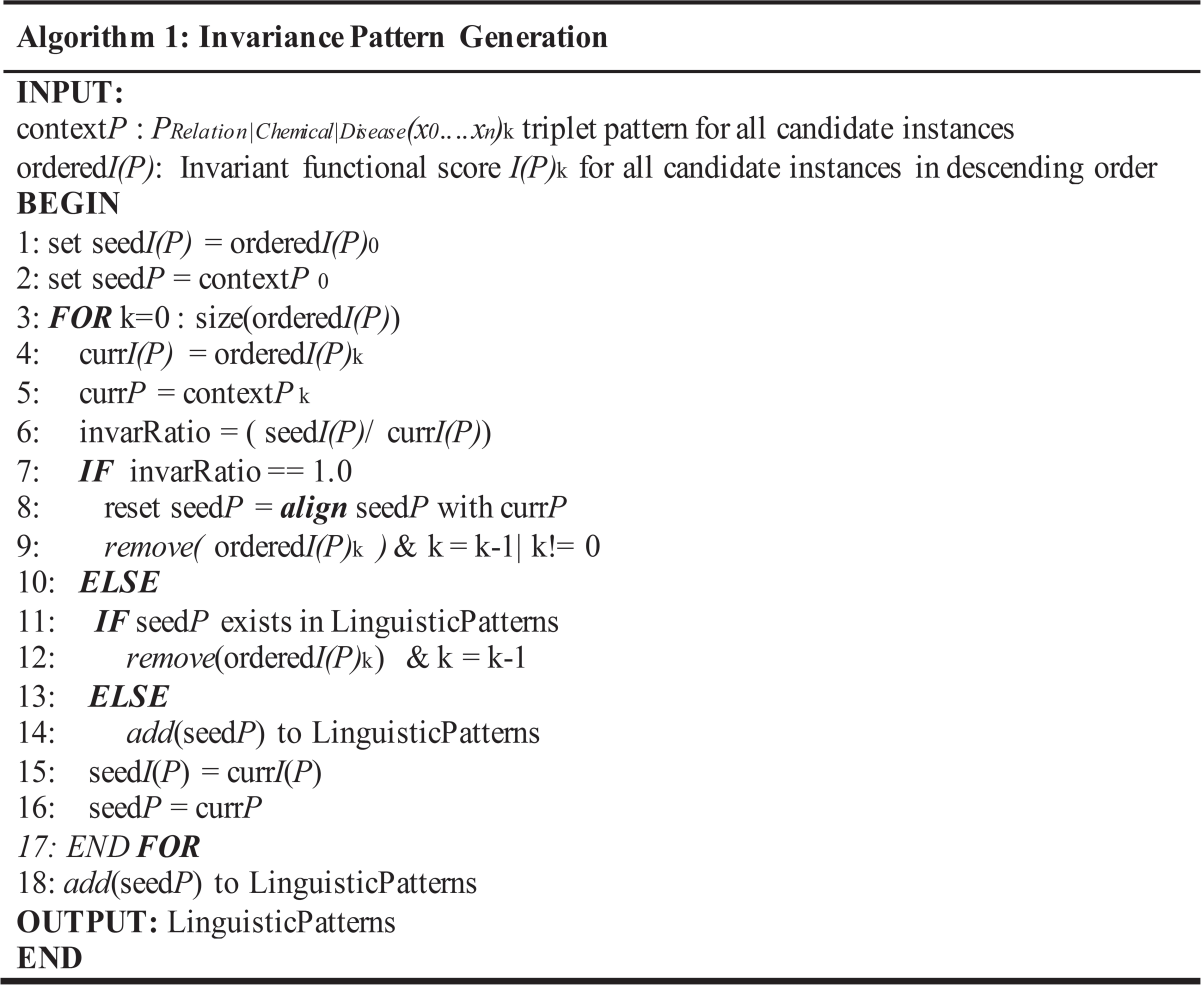
Algorithm for invariance-based linguistic pattern identification.

**Figure 7:**
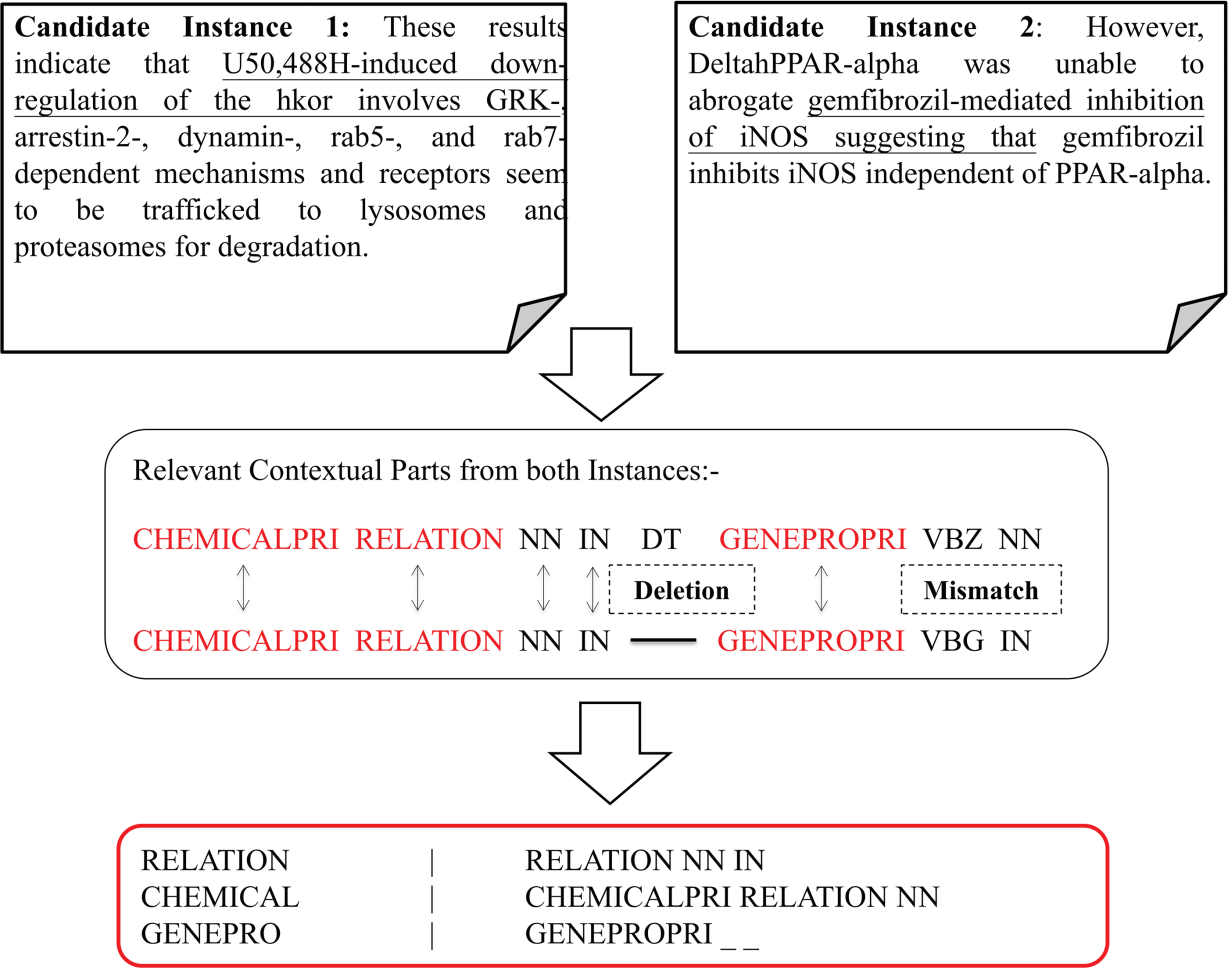
Context identification and prospective alignment of candidate instances.

Modeled according to (3), each candidate instance shapes as a conic section in Cartesian space based on its PoS-Tag-based polynomial representation identifying its invariant score *I(p)* shown in Figure 5. The ratio of their invariant scores is treated as the discriminative property to determine the respective sentient interpretations as similar or otherwise. Therefore PoS-Tag instances are ranked in the descending order based on respective *I(p)*_k_ scores and pre-initialized parameters (Δ = 1.00 and W∼1) from (2). Each candidate instance is compared with its successor and if the ratio of scores = 1, instances are considered structurally similar and clustered together to form a linguistic pattern for classification. Otherwise, they are diversified into different groups as communicated by Figure 6. Δ = 1.0 impresses that we preset the condition of minimal rotation (*ф* = 0) between instances. Structurally similar PoS-Tag-based contexts attain similar *I(p)*_k_ scores and therefore invariance ratio equal to 1.0 between instances. By contrast, dissimilar PoS-Tag contexts have significantly different *I(p)*_k_ scores due to contextual variance, registering invariance ratio ≠ 1.0 that signifies inferential divergence between instances. *W* = 1 highlights that instances are measured as absolute representations opposed to a factor of their transformations. Similar invariance instance clusters are subjected to optimization via alignment per group to generate a triple context set-based pattern as shown in Figure 7. Within each relation type, multiple patterns are obtained showcasing diverse permutations of PoS descriptions with inferentially same interaction type. The alignment optimization is based on the highest scoring path obtained from the substitution matrix delivered by the recurrence relation given below:

(6) \begin{equation*} sim\left(i,j\right)=\left\{\begin{array}{c}\left\langle sim\left(i,j-1\right)+\lambda \left(\_,j\right)\approx -2\right\rangle \\ \!\!\! \left\langle sim\left(i-1,j-1\right)+\lambda \left(i,j\right)\approx \left|\begin{array}{c}2\forall i=j\\ {}-2\forall i\ne j\end{array}\right.\right\rangle \\ {}\left\langle sim\left(i-1,j\right)+\lambda \left(i,\_\right)\approx -2\right\rangle \end{array}\right.\!\!\!, \end{equation*}

where *sim(i,j)* is the similarity score of the *i^th^* row and *j^th^* column of the substitution matrix. *λ(i,j)* indicates the penalty function scoring insertion, deletion, match or mismatch depending on the token comparison of respective indices. The triplet of each pattern represents immutable linguistic segments from each cluster of inferentially similar instances.

**Figure 8:**
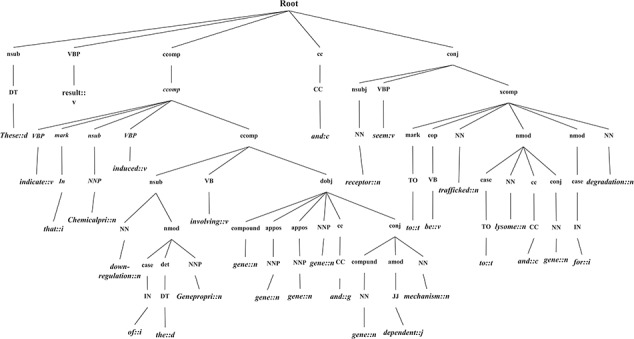
GRCT representation of a candidate instance.

### Linguistic pattern-aware dependency tree construction

**Figure 9:**
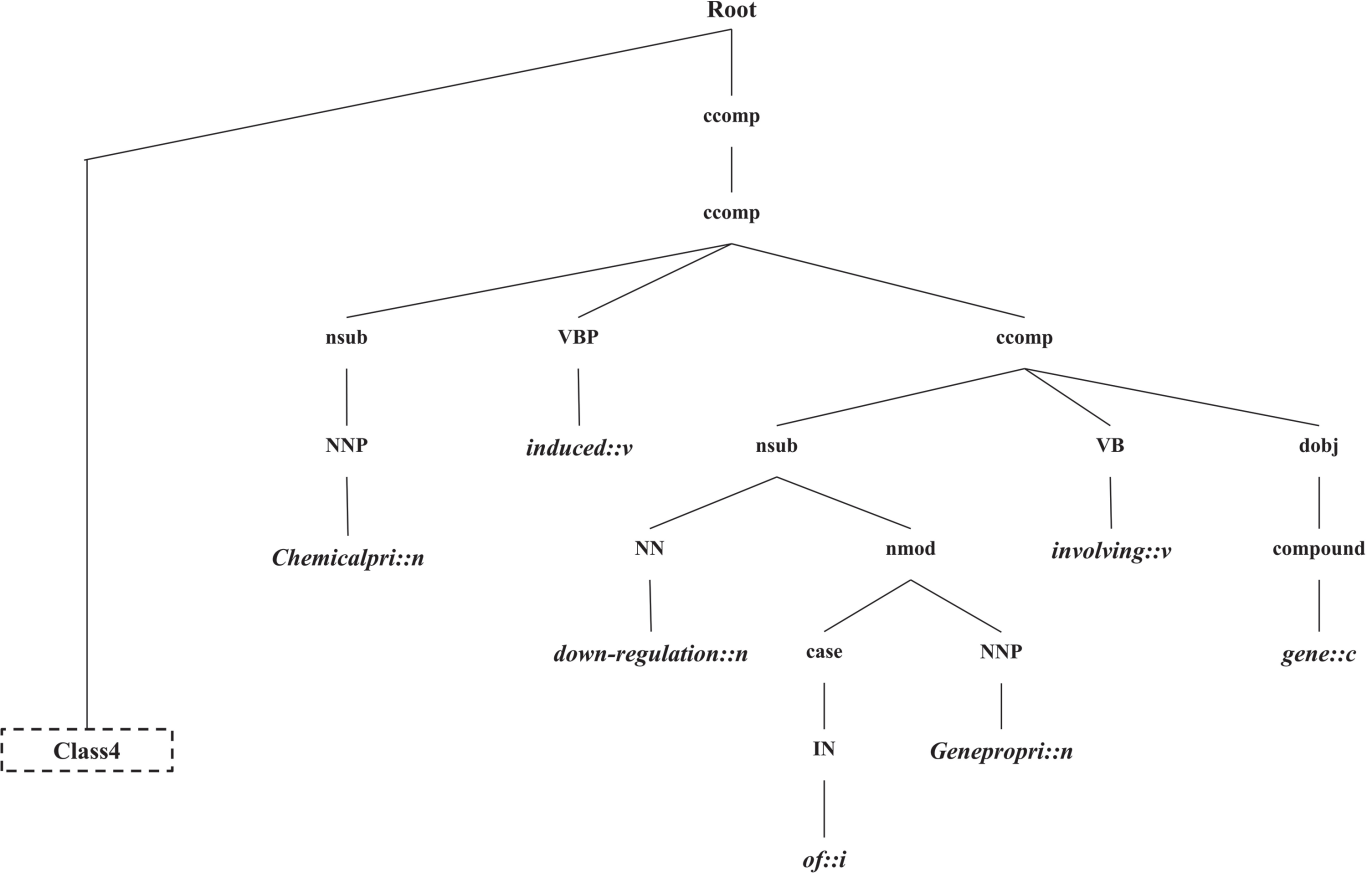
The generated CPI tree.

In this section, we introduce the candidate instance-based linguistic pattern-aware dependency tree structure, which is built on grammatical relation centered tree (GRCT) (6) enhanced by pruning and decoration operations on sentences. To facilitate comprehension, we exemplify the process of tree construction using the sentence `These results indicate that U50,488H-induced down-regulation of the hkor involves *GRK-*, *arrestin-2-, dynamin-, rab5-* and *rab7*-dependent mechanisms and receptors seem to be trafficked to lysosomes and proteasomes for degradation’. Figure 8 displays the GRCT representation of this candidate instance. It adds tags of grammatical relations and lexical information as new nodes in the constituency tree, emphasizing enhancement of lexical information by adding grammatical relations and PoS-Tags as the rightmost children. Therefore, in contrast to shallow parsed trees, GRCT generates further layers of relevant information for the tree kernel to determine similarity. The PoS-Tag labels referenced in the consolidated tree structure align more conveniently with our feature enriching strategy of linguistic patterns, making it an ideal option in this implementation. The initial parse tree used for enhanced GRCT representation remains as the Stanford parser (4, 24, 36). Hence generated tree structures often comprise of lexical components, which holistically bring grammatical coherence, but may compound as unnecessary noise for statistical classification. We aim to target this selective noise within the tree structure by pruning parts of the tree using linguistic patterns from Invariance-based feature optimization section. These stringent context patterns are aligned with the PoS-Tag representation of the GRCT based candidate instance. The optimal and best scoring matched pattern is selected to prune the GRCT representation-based candidate instance for learning optimized features. The pruning operation removes the leaf nodes and their preceding hierarchies that were not contained in the pattern, thereby bringing brevity and more discriminative power to the tree for classification. To refine the tree structure of interacting entities from non-interacting ones, additional decoration is undertaken by highlighting the instances with a prefix node denoting their respective interaction class (2, 3). These operations are elucidated in Figure 9, depicting how most of the noisy tree is trimmed by the selected linguistic pattern to keep only chemicals, relation and protein-associated contexts in the final feature. An additional CPR tag is added to distinguish the association type and favor the tree from non-interacting entity trees during classification e.g Class 4 (representing CPR4-type interaction) as shown in Figure 9. During the construction of feature trees for test case instances, we observe an additional optimization by using an interaction keyword-based filter. These keywords are the proximal verbs derived from the training data sets.

SVM kernel is used to classify the tree-based features. In SVM, a kernel function can efficiently compute the similarity between two instances without identification of the entire feature space. In the case of a tree kernel, features are described using tree substructures to evaluate the number of common tree fragments between two trees *T_1_* and *T_2_* through the following equation:

(7) \begin{equation*} K\left({T}_1,{T}_2\right)={\sum}_{n_1\in {N}_{T_1}}{\sum}_{n_2\in {N}_{T_2}}\Delta \left({n}_1,{n}_2\right), \end{equation*}

where ${N}_{T_1}$ and ${N}_{T_2}$ denote the sets of nodes in *T_1_* and *T_2_*, respectively. The function ∆(*n*_1_, *n*_2_) signifies the number of common fragments rooted in *n_1_* and *n_2_* nodes. The proportional variation in number of different sub-trees versus the original parse tree size makes them computationally unsuitable to be reproduced as feature vector.

Therefore, in order to tackle this complexity issue, multiple tree kernels have been proposed in recent years such as syntactic tree kernel (28, 39), partial tree kernel (30, 31) and lexical semantic kernel (37). However, the lexical part in these tree kernels comprises of leaf nodes with exactly same structures limiting its applications. Croce *et al*. (7) proposed a much more general SPTK that can be applied to any tree and exploit any combination of lexical similarities with respect to the syntax enforced by the tree. Therefore, we adopted SPTK to capture the syntactic similarity between the high-dimensional vectors implicitly as well as partial lexical similarity of the trees. The *∆ _SPTK_* (*n*_1_, *n*_2_) can be defined as follows.

If nodes *n_1_* and *n_2_* are leaves, then *∆ _SPTK_* (*n*_1_, *n*_2_) *= μλσ*(*n*_1_, *n*_2_).

Otherwise, calculate *∆ _SPTK_* (*n*_1_, *n*_2_) recursively as

(8) \begin{equation*} {\displaystyle \begin{array}{l}{\Delta}_{\sigma}\left({n}_1,{n}_2\right)=\\ {}\mu \sigma \left({n}_1,{n}_2\right)\times \Big({\lambda}^2+\sum \limits_{{\vec{I}}_1,{\vec{I}}_2,l\left({\vec{I}}_1\right)=l\left({\overrightarrow{I}}_2\right)}{\lambda}^{d\left({\vec{I}}_1\right)+d\left({\vec{I}}_2\right)}\times \\ {}\prod \limits_{j=1}^{l\left({\vec{I}}_1\right)}{\Delta}_{\sigma}\left({c}_{n1}\left({\vec{I}}_{1j}\right),{c}_{n2}\left({\vec{I}}_{2j}\right)\right)\Big),\end{array}} \end{equation*}

where *σ* is any similarity between nodes and *μ*, *λ*∈[0,1] are two decay factors. ${\vec{I}}_1$ and ${\vec{I}}_2$ are two sequences of indices that index subsequences of children *u*, $\vec{I}=\left({i}_1,\dots, {i}_{\left|u\right|}\right)$, and in sequences of children *s*, $1\le {i}_1<\dots <{i}_{\left|u\right|}\le \left|s\right|$, i.e. such that $u={s}_{i1}\dots {s}_{i\left|u\right|}$, and $d\left(\vec{I}\right)={i}_{\left|u\right|}-{i}_1+1$ is the distance between the first and last child. $c$ is one of the children of the node $n$ that is also indexed by $\overset{\rightharpoonup }{I}$. This provides an advantage that tree fragments can be matched by applying the embedding similarity *σ*, even if these tree fragments are not identical but are semantically related.

## Experiments

### Experimental setup

Our focus in approaching this task of bio-entity relation identification was to reinvent parts of feature representation and study its impact as a factor in improving classification results. Therefore, we designed our experiments with different biological corpora aimed at the task of relation identification. In the first set of experiments we used CHEMPROT corpus released under BioCreative VI to identify specific types of interaction between chemical and protein. Keeping in context the interaction-based study, we adapted CDR corpus from BioCreative V for the second set of experiments. Lastly, we employed LPTK approach in studying PPIs from five PPI corpora. Each of these tasks lends aid in understanding different biological interactions, which are systematically related to the holistic implementation of precision medicine. Each study was evaluated on metrics of precision, recall and F_1_-score (25), as well as the micro-average used for comparing the average performance. These measures are defined based on a contingency table of predictions for a target category *C_n_*. Precision *P*(*C_n_*), recall *R*(*C_n_*), F_1_-score *F_1_*(*C_n_*) and micro-average *avgμ* are defined as follows:

(9) \begin{equation*} P\left({C}_n\right)=\frac{TP\left({C}_n\right)}{TP\left({C}_n\right)+ FP\left({C}_n\right)} \end{equation*}

(10) \begin{equation*} R\left({C}_n\right)=\frac{TP\left({C}_n\right)}{TP\left({C}_n\right)+ FN\left({C}_n\right)} \end{equation*}

(11) \begin{equation*} {F}_1\left({C}_n\right)=\frac{2\times P\left({C}_n\right)\times R\left({C}_n\right)}{P\left({C}_n\right)+R\left({C}_n\right)} \end{equation*}

(12) \begin{equation*} {avg}_{\mu}\left({C}_n\right)=\frac{\sum_{k=1}^l{F}_1{\left({C}_n\right)}_k\times \widehat{w}{\left({C}_n\right)}_k}{\sum_{k=1}^l\widehat{w}{\left({C}_n\right)}_k}, \end{equation*}

where *TP*(*C_n_*) denotes the number of true positives (number of positive instances that are correctly classified), and *FP*(*C_n_*) denotes the number of false positives (FP) (instances which are negative instances that are erroneously classified as positives). Analogously, *TN*(*C_n_*) and *FN*(*C_n_*) stand for the number of true negatives and false negatives (FN), respectively. The F_1_ value is used to determine the relative effectiveness of the compared methods. Micro-average is gathered using weight-averaged F_1_ score for each evaluation set, weighted with proportional data size ${w}_k$ per test data set.

**Table 3:** Distribution of the CHEMPROT data set (http://www.biocreative.org/resources/corpora/chemprot-corpus-biocreative-vi/)

**Data set**	**#Abstract**	**#Chemical**	**#Gene/protein**	**#Relation**
Training	1020	13 017	12 735	4157
Development	612	8004	7563	2416
Test	800	10 810	10 018	3458

**Table 4:** Descriptive statistics of the CDR data set (http://tinyurl.com/pubtator-cdr)

**Data set**	**#Abstract**	**#Chemical**	**#Disease**	**#Relation**
Train	500	4182	5203	1038
Development	500	4244	5347	1012
Test	500	4424	5385	1066

### Data set

#### CHEMPROT data set

The CHEMPROT data set provided by the CPI task–BioCreative VI for separate phases varies in abstract size and corresponding relation measures as depicted in Table 3. The training data comprises of 1020 abstracts with 4157 annotated relations. Corresponding chemical and protein entity annotations are provided with the corpus and tagged with generic IDs such as `Arg 1’. We initially reported our results with the test data encompassing 3399 abstracts, out of which only 800 have complete annotations. This experiment uses toolkits from Apache Open NLP and GENIA along with our entity-normalization module for feature optimization. The window size for pattern optimization is adjusted to *n* = 3. We use SPTK classifier for this multi-class task resourcing libraries from the KeLP (http://www.kelp-ml.org) platform (11). Due to its multi-class nature, we only screen for positive interaction labels (i.e. CPR3, CPR4, CPR5, CPR6 and CPR9) using classifier. Based on the development data, prediction cut-off for positive and negative test cases is adjusted to >0 and −0.3, respectively. Highest score from class prediction results is use to label entity-pair interactions.

#### CDR data set

The CDR corpus consists of 1500 abstracts from PubMed, which are evenly distributed among the training, test and development data sets as shown in Table 4. Chemical and disease annotations are marked with respective MeSH ID as a part of the corpus, and entities are normalized to their respective type forms. Due to the similarity in nature of the tasks, we furnish the corpus with the same preprocessing pipeline mentioned earlier using Apache Open NLP (http://opennlp.sourceforge.net/models-1.5/en-sent.bin) and GENIA (http://www.nactem.ac.uk/tsujii/GENIA/tagger/geniatagger-3.0.2.tar.gz) toolkit followed by invariance-based feature optimization. The frame size for optimization is restricted to *n* = 5. This task is a binary classification problem, as it needs to identify whether respective heterogeneous entities are involved in an interaction or not. Each test instance is duplicated for both cases (+*ve*/−*ve*) and then evaluated against the training model, with the higher score as determinant of the instance label.

#### PPI data set

For PPI experiments, we have used five PPI corpora (40) available in public domain detailed in Table 5. Each of the databases is different in size, with HPRD50 having the least number of abstracts [50] and BioInfer with the most number of abstracts [1100]. Corresponding protein entity annotations are provided with the corpus and tagged with generic IDs such as `T1’. Each corpus is evaluated separately using the entire set in 10-fold cross validation. The same preprocessing toolkit described in Candidate instance generation section is used. For pattern optimization, the window size is settled at *n* = 3. SPTK classifier is employed in this binary classification task resourced from the KeLP platform. Entity pairs are labeled as interacting pairs if the prediction score is within the range of 0–1.0.

**Table 5:** Statistical distribution in five PPI corpora (http://corpora.informatik.hu-berlin.de)

**Data set**	**#Abstract**	**#Sentence**	**#POSITIVE**
HPRD50	50	145	163
LLL	77	77	164
IEPA	486	486	335
AIMed	225	1955	1000
BioInfer	1100	1100	4834

### Results

#### CPI task performance

Table 6 is a summary of our results on training, development and test data set performances in both binary and multi-class setting. Relation pairs in each test case are based on the combination of all pre-annotated entity pairs described for each instance of the interaction class. We evaluate our results at two levels (binary and multi-class) to study the efficiency offered by the linguistic pattern-based dependency tree kernel. Our system achieves F_1_-score of 47.58% on test data identifying interacting versus non-interacting entity pairs with a recall rate of 71.80% for the ChemProt pair task. The F_1_-score dips to 36.54% screening for specific relation interaction within predicted interacting classes in test set while the recall reduces to 55.14%. Results from training, development and test data conclude a consistent 9% drop in F_1_-score metric between binary and multi-class classification.

**Table 6:** Performance evaluation on the CHEMPROT corpus for different classification tasks

**Data set**	**Classification type**	**Task type**	**Precision (%)**	**Recall (%)**	**F**_**1**_ **-score (%)**
Training	Binary	Chemical–protein pair	36.90	75.79	49.64
	Multi-class	CPR	30.11	61.84	40.50
Development	Binary	Chemical–protein pair	33.69	71.68	45.84
	Multi-class	CPR	25.83	54.96	35.15
Test	Binary	Chemical–protein pair	35.58	71.80	47.58
	Multi-class	CPR	27.32	55.14	36.54

Our multi-class classification strategy focuses on harnessing class deterministic features to stabilize the dependency tree kernel-based classification method. Table 7 lists the class-wise performance of our system on three of the relation types. There is a gradation in the relative performance among various classes with 43.43% F_1_-score for CPR4. The linguistic pattern-based tree kernel recognizes ∼80% of the entity pairs correctly from CPR4, followed by a recall of 35.83% for CPR6. This steep bias across the category-based recognition is partly due to the imbalanced size of candidate instances for each of the relation type in both the training and the test data set as elucidated in Table 2. This imbalance subsequently impacts feature pattern representation generated for the corresponding class and their subsequent cross-class optimization. This is because the linguistic patterns are derived from a word frame-based probabilistic model calculated on the candidate instances for each class separately. More diverse and sizeable candidate instances for each class ensure comprehensive and robust linguistic features correspondingly. A relatively reduced number of instances from any class converge the size of corresponding feature patterns and may also affect the cross-class feature optimization if pattern representation becomes too generic. During the training process this bias of the linguistic patterns transcends to the tree kernel, causing the current class against the rest (OvA) to become uneven due to the limited representation size.

**Table 7:** Relative variations in performance for each CPR task

**Relation type**	**Precision** (%)	**Recall** (%)	**F**_**1**_ **-score** (%)
CPR 4	29.74	80.43	43.43
CPR 6	37.10	35.83	36.45
CPR 5	19.54	48.20	27.81

Table 8 displays a comparative analysis of the system results on the ChemProt task of BioCreative VI. Due to the multi-class nature of the task, many teams opted to use an ensemble system by grouping different kernels to secure enriched feature results from each stage. For instance, Lung *et al*. (21) built a three-stage model using semantic information and dependency graph to develop paired and triplet entity relation features achieving F_1_-score of 56.71%. Tripodi *et al*. (41) employed various classifiers with the distinction of using external KBs incentivized features. Furthermore, Wang *et al*. (47) used a four-layered LSTM model consisting of feature layers comprising distances of chunked entity pairs to restructure hidden knowledge. They obtained an average performance of 38.39% with recall at 66%. As part of the deep learning method groups, Yuskel *et al*. (46) used a CNN—word-embedding-based learning model, which acquires a performance of 18.64%. In addition to the method-segmented comparison, we compared our performance to the registered baseline based on entity CO, which scored 0.99 and 8.37% for abstract and sentence-level classification, respectively. An overall comparative assessment of the methodologies suggests a mixed bag where singular kernels (including deep learning methods) take lower spots as opposed to the superior ensemble kernels. The layered structure of ensemble methods brings in the distinction from other methods as each sieve refines multiple classifier features to further accuracy.

**Table 8:** Comparison of the system performances on the CPI task

**Classifier strategy**	**System**	**Precision** (%)	**Recall** (%)	**F**_**1**_ **-score** (%)
Baseline (CO)	Abstract	0.50	100	0.99
	Sentence	4.37	98.03	8.37
Ensemble kernels	Lung *et al*. (21)	63.52	51.21	**56.71**
	Tripodi *et al*. (41)	33.87	40.78	37.00
RNN-LSTM	Matos *et al*. (27)	57.38	47.22	51.81
	Wang *et al*. (47)	26.96	**66.63**	38.39
CNN	Yuksel *et al*. (46)	60.57	11.02	18.64
Tree kernel	Warikoo *et al*. (49)	29.32	32.71	30.92
	Our method	27.32	**55.14**	36.54

Our system was the only model in this challenge where tree kernel-based approach was used in resolving relation interaction. Therefore, we also performed additional experiments to study how the generated linguistic pattern impacted tree representation classification by comparing the overall performance of our representation model against other popular tree representation based on grammatical and lexical properties.

As shown in Table 9, we studied performances of various tree representations, such as GRCT, Compositional Grammatical Relation Centered Tree (CGRCT), Lexical Centered Tree (LCT), Compositional Lexical Centered Tree (CLCT) and Lexical Only Centered Tree (LOCT) against LPTK (6, 32). Both binary and multi-class classification reported an average jump of 12 and 6% in performance, respectively, using LPTK representations. In addition, the training time required in learning from LPTK representation also proved to be consistently lower than most of the other compared tree representations. The overall performance of non-LPTK representations is more or less similar to each other in both binary and multi-class tasks. An exception to this trend is LOCT, whose performance fares relatively better, averaging to 41.54% in binary classification. LOCT also achieved 35.42% in multi-class classification; nevertheless, it still lags behind in relative performance achieved by LPTK model.

**Table 9:** Comparative assessment of LPTK representation against other tree representations

**Tree representation**	**Training time (sec)**	**Classification type**	**Precision (%)**	**Recall (%)**	**F**_**1**_ **-score (%)**
GRCT only	10888	Binary	35.51	35.5	35.50
		Multi-class	27.64	27.63	27.64
CGRCT only	6831	Binary	27.41	43.32	33.58
		Multi-class	24.69	39.03	30.25
LCT only	5871	Binary	32.27	41.5	36.31
		Multi-class	27.99	35.99	31.49
CLCT only	7104	Binary	46.26	22.75	30.50
		Multi-class	41.09	20.21	27.09
LOCT only	605	Binary	40.06	43.14	41.54
		Multi-class	34.15	36.78	35.42
LPTK	629	Binary	35.58	71.8	**47.58**
		Multi-class	27.32	55.14	**36.54**

#### CID task performance

Table 10 displays the performance of our system with an F_1_-score of 55.18% on the CID task. The use of optimized features in learning the model has a positive effect on the system ability to screen true positive associations effectively, as our method registers one of the highest recalls reported on the data set to date. Other popular approaches used in this task rely mostly on external KB supported features like entity-pair provenance and mentions in databases. The performances range from 41 to 70%, with Pons *et al*. (34) being the top performer in the group. They leveraged information from the Euretos and CTD repository to enhance weight bias for the interacting entity pairs gaining ∼12% increase over their previous performance. Xu *et* al. (44) enlisted SIDER, CTD and MeSH databases to enhance the contribution of lexical features related to known entity pairs. Based on their experiments, entity and lexical features result in a performance of 50.73%, with significant improvements reported only upon the addition of external KBs propelling F_1_-score to 67.16%.

**Table 10:** Comparative assessment of system performances on the CID task

**Method**	**System**	**Description**	**Precision** (%)	**Recall** (%)	**F**_**1**_ **-score** (%)
External KB adjusted features	Li *et al*. (23)	Lexical + SVM	54.46	33.21	41.26
	Pons *et al*. (34)	Graph + SVM	73.10	67.60	70.20
	Alam *et al*. (51)	Feature + SVM	43.68	80.39	56.61
	Xu *et al*. (44)	KB + Lexical + SVM	65.80	68.60	67.20
Optimized lexical features	Le *et al*. (52)	Lexical + SVM	53.41	49.91	51.60
	Gu *et al*. (13)	CNN + ME + PP	**55.70**	68.10	**61.30**
	CoSine	Cosine similarity features + CTK	44.41	58.91	50.64
	Our method	LPTK	39.28	**92.68**	55.18

**Table 11:** Comparative assessment of our method with other state-of-the art approaches in PPI task

**Method**	**System**		**HPRD50**	**LLL**	**IEPA**	**AIMed**	**BioInfer**	**avg**_**μ**_
			*Precision (P), Recall (R), F_1_-score (F_1_) [%]*					
Feature-based	CO	P	38.9	55.9	40.8	17.8	26.6	25.2
		R	100	100	100	100	100	100
		F_1_	55.4	70.3	57.6	30.1	41.7	40.2
Multi-kernel	Miwaa *et al*. (29) (CK)	P	68.5	77.6	67.5	55.0	65.7	62.4
		R	76.1	86.0	78.6	68.8	71.1	71.1
		F_1_	70.9	80.1	71.7	60.8	68.1	66.5
	Airola *et al*. (1) (GK)	P	64.3	72.5	69.6	52.9	56.7	56.5
		R	65.8	87.2	82.7	61.8	67.2	66.4
		F_1_	63.4	76.8	**75.1**	56.4	61.3	61.1
Tree kernel	Satre *et al*. (38) (AkanePPI)	P	52.0	76.7	66.2	29.1	56.8	48.1
		R	55.8	40.2	51.3	52.9	85.4	71.0
		F_1_	53.8	52.8	57.8	37.5	68.2	57.3
	Chang *et al*. (2) (PIPE)	P	63.8	73.2	62.5	57.2	68.6	64.4
		R	81.2	89.6	83.3	64.5	70.3	69.6
		F_1_	**71.5**	**80.6**	71.4	60.6	66.5	66.9
	Our method (LPTK)	P	72.7	78.9	74.8	83.6	83.0	81.6
		R	62.2	72.1	66.1	75.0	67.5	68.9
		F_1_	67.1	75.3	70.2	**79.1**	**74.4**	**74.7**

In comparison to these methods, other systems strictly implement learning without employing external KBs. One such system by Gu *et al*. (13) used a multi-kernel method attaining 61.30% on F_1_-score. They utilize ME and CNN classifiers to tackle inter- and intra-sentence level entity classification.

#### PPI performance

PPI is one of the vastly documented biological interactions based on scientific literatures compiled from multiple corpora. Table 11 presents a summary of our results by evaluating the LPTK approach on these five different PPI corpora. Our system performed well on relatively bigger PPI corpora, i.e. IEPA, AIMed and BioInfer with each recording the F_1_-score of 70.2, 79.1 and 74.4%, respectively. Significant precision and recall measures from each corpus reflect the effectiveness of linguistic patterns in representing distinctive learning features within PPIs. Various models have been used earlier to resolve the PPI task. Airola *et al*. (1) worked on a feature-based approach using entity CO within sentences in dependency graph representation. This method fared particularly well in small sized corpus *viz.* LLL reaching F_1_-score of 70.3%. Other popular approaches in PPI task included multi-kernel approach where features extracted from multiple kernels were combined to determine interactions as shown in the works of Miwaa *et al*. (29). They exploited layers of syntactic structures from sentences using different parsers to build a composite kernel (CK) for PPI extraction. Their system performed well on all five PPI corpora with ${F}_{\mu }$ = 66.5. Using a similar approach, Airola (1) explored the subgraph and linear order subgraph parse structures to build an all-path GK. Results indicate that the multi-kernel methods significantly outperformed the feature-based approaches.

Among the other kernel approaches, syntactic tree structures have also been immensely implemented in classifier kernels for PPI extraction. One of the earliest consolidated tree kernel approaches in PPI extraction was the AkanePPI introduced by Satre *et al*. (38), which is a system featured on shallow dependency parsers combined with semantic features from deep syntactic parsers. This system obtained a good F_1_-score of 68.2% with the BioInfer data set. Chang *et al.* (2) developed PIPE, which is an interaction pattern tree kernel approach integrating PPI-patterns with the convolution tree kernel. It utilizes features consisting of interaction patterns unlike any of the previous methods to achieve a competent score compared to the multi-kernel approaches. PIPE performed well on the five PPI corpora, registering a 66.9% ${F}_{\mu }$. As one of the tree kernel-based approaches, our method employed more elaborative linguistic patterns for feature representation and exhibited improved overall performance on all of the PPI corpora with an average ${F}_{\mu }$ = 74.7%.

### Discussion

#### CPR result analysis

LPTK is an entirely statistical learning-based approach. With CPR experiments, we attempt to explore a new method effective on association identification by using singular tree kernel classifier enriched with linguistic patterns. We have performed different experiments and levels of comparison to inspect the contributions of linguistic patterns in generating distinctive tree representations. The experiments with tree representation performance of LPTK in Table 9 showed that linguistic pattern-based tree representations are efficient and robust. Singular feature representations based on GRCT perform poorly compared to LPTK in both binary and multi-class interaction identification between chemical and protein. LPTK employs linguistic pattern, which prunes and decorates the GRCT representations, registering a jump of ∼12 and 9% in binary and multi-class scenario tasks against standalone GRCT representation, respectively. This shift in performance within the same tree representation over enhancements via linguistic patterns endorses the contribution of linguistic patterns in generating effective learning representations. The linguistic patterns generated based on feature invariance are more effective in removing the noisy segments from the sentences while preserving the most relevant contexts for semantic relation analysis as shown in Figure 9. On the contrary, the standalone tree representations employ the entire deep parser-based representation, which in many instances leads to generic sub-tree representations, causing performance to drop with partial tree kernels. LOCT performs relatively well in this scenario, as the sub-tree structures are lexicon-centric and do not include repetitive grammatical sub-structures. For instance, the LOCT representation of the instance `Phosphatidylserine (PtdSer) is made in mammalian cells by two PtdSer synthases, PSS1 and PSS2’ is given by [(#be::v(#chemical::n(#chemicalpri::n))(#make::v(#cell::n(#in::i)(#mammalian::j))(#two::c(#by::i)(#genepropri::n(#,::,)(#genepro::n)(#and::c)(#genepro::n)))))]. Evident from the representation, the sub-tree structures aren’t deep enough which co-incidentally aid in classifying trees based on lexicons directly leading to a relative increase in the performance compared to other standalone representations. Most of the standalone tree representations in this experiment occur in more or less similar bandwidth of performance except LPTK, which scores relatively better on both F_1_-score and training time.

Though results from Table 6 highlight a structural weakness in the patterns to effectively screen out FP within CPI task. Based on further analysis, we attribute the low precision in CPR task to be a combined factor of normalized relation terms and lesser weight distributed to leaf nodes by the dependency tree kernel. For example in instance `Activation of endothelial nitric oxide synthase (eNOS) results in the production of nitric oxide (NO) that mediates the vasorelaxing properties of endothelial cells.’ word frames based on underlined key terms are compared and evaluated against PoS-Tag pattern. At the classifier level, SPTK gives weight bias to upper nodes (PoS-Tags) in the dependency tree versus the terminal nodes (actual word). Since the PoS-Tags label is ranked higher in the GRCT structure, it normalizes the differentiating power that could be attained by the relation verb word at the higher level. After further investigation, we conclude that the problem cannot be mitigated by rearranging the tree structure alternating text on top, as this would result in less frequent word sub-trees to be recognized as outliers and significantly impairing the recall.

During our experiments with multi-class classification in CPR task, we observed that the efficiency in identifying some of the relation classes was relatively lower than the others. As shown in Table 7, the CPR6 and CPR5 interaction classes were the ones that were extremely difficult to identify. As discussed earlier, these classes have far fewer representations in the corpus, which adversely impacts the quality of information rendered by the corresponding linguistic patterns to distinguish them from rest. Take the example of following test candidate instance 1: `Astemizole, a potent histamine H1-receptor antagonist: effect in allergic rhinoconjunctivitis, on antigen and histamine induced skin weal responses and relationship to serum levels’ and instance 2: `Amitriptyline is aTrkA and TrkB receptor agonist that promotes TrkA/TrkB hetrodimerization and has potent neurotropic activity’. The underlined section dispenses the main context of relations in both cases, which seem to be linguistically similar up to a certain extent. Distinct tree representations for these sentences are refined by possible patterns, including pattern1: [CHEMICAL DT JJ ➔ GENE NN. ➔ RELATION. .] and pattern2: [CHEMICAL VBZ DT ➔ GENE. . ➔ RELATION. .] from CPR6 and CPR5, respectively. These patterns are analogous in certain contexts (*viz.* relation-based and gene-based contexts can be respectively aligned), and their subsequent tree representations are also comparable in length and tagged labels. This similarity in representation leads to the misclassification of instance1 as `CPR5’ by the partial tree kernel, while it actually belongs to `CPR6’. These cross-category tree representations resulted in FP and FN within CPR task, thereby decreasing the class-wise and average performance. In the future, we intend to enhance the optimization of linguistic patterns with dynamic context size to reduce outlier errors and manage hyperplane boundaries.

It is noteworthy that our method is the only tree kernel-based approach implemented in this challenge with performance comparable to some of the ensemble systems as well as the deep learning approaches as is evident from Table 8. We fall short against multi-feature-based layered kernels, as our classifier features are contingent on singular parameter of context patterns. Diversification of features can help us accomplish a close comparison. In spite of this, our approach is quite unique and universal. The features generated by our invariant learning forms are human-readable and can be reused on corpora of similar context. These extensive patterns help us obtain a recall of 55.14% that is higher than most of the other systems. Representational convenience of these patterns can be exhibited by the following example. For instance, the generated pattern [CHEMICAL RELATION DT ➔ RELATION DT NN ➔ GENE NN NN] identifies the relevant context from the instance `We find that FP causes a decrease in stimulated eosinophil secretion of LTC4 that is regulated by phospholipase A2’; trying to assert that `FP’ causes decrease in the `phospholipase A2’ regulatory activity while pruning any noisy tokens brought in by additional entities.

In contrast to abstract representational features added in deep learning methods, linguistic patterns are explicit, simple and robust. By maintaining the same representational features, we experimented with the nature of tree kernels used for CPI extraction. We upgraded our kernel to SPTK as opposed to CTK used in the BioCreative VI submissions (49), and the kernel modification brought in marginal performance improvement with ∼22% increase in the recall.

#### CID result analysis

Based on the results displayed in Table 10 the performance of LPTK in CID task may not be at par with KB-based methods but it comes a close second against deep learning-based hybrid systems giving a promising recall of 92.68%. We credit this to the extensive automated patterns generated by LPTK that can match a variety of linguistic contexts under different inferential scenarios described in CID data set. For example, in the instance `*Famotidine associated delirium*’, a simple pattern of [CHEMICAL ➔ RELATION ➔ DISEASE] is sufficient to identify relation, while in case of a more elaborate instance like `The use of thiopentone was significantly associated with an eight-fold-higher risk for delirium compared to propofol (57.1 % versus 7.1 %, RR = 8.0, χ (2) = 4.256; df = 1; 0.05 < *P* < 0.02*).*’ it requires further descriptive linguistic pattern [DT NN IN CHEMICAL➔ IN CHEMICAL _ RELATION ➔ _ _ DISEASE] to determine the associative relation. These patterns vary in size and contextual structure owing to their consolidation upon the training set. Our automated linguistic patterns are methodical in summarizing the significant inferential bits from each sentence based upon clustered information contexts rather than randomly aligning lexical segments against each other. This demonstrates the true power of invariance-based linguistic patterns and explains their ability to predict relational inferences with greater efficacy. The comparative analysis in Table 10 showcases our method as a novel system, which gathers a wider spectrum of auto-generated features to reduce the dependency of classifiers on manually enriched features largely contingent on external KBs.

#### PPI result analysis

In addition to the CPI and CID tasks, we included the experiment results of PPI extraction to showcase the effectiveness of LPTK in recognizing other biological entity relations like PPI. Based on the results in Table 11, our method outperforms the previously listed methods on at least 2 out of 5 corpora, showing promising performances of 79.1% for AIMed and 74.7% for BioInfer, respectively. These results exhibited a significant boost from the previously reported best performances for both data sets (19% for AIMed and 6% for BioInfer). Our analysis revealed that the size and information specific content of PPI data sets are attributive to the performance improvement. As referenced previously, LPTK is based on statistically significant patterns and the extensiveness of these corpora makes it conducive to retrieve empirical patterns of interaction. For instance, in the sentence `All these results suggest that cofilin is a new type of actin-associated protein.’, key terms `cofilin’ and `actin’ are related by a type categorization. In another sentence from a different abstract `We conclude that *Aip1p* is a cofilin-associated protein that enhances filament disassembly activity of cofilin and restricts cofilin localization to cortical actin patches.’ another set of key terms *viz. Aip1p* and `*cofili*’ are again associated by type categorization. Due to their contextual similarity, invariant method identifies the same linguistic pattern representation [RELATION IN PROTEINAGENT ➔ PROTEINAGENT VBZ DT ➔ PROTEINTARGET VBN PROTEIN] for them. By contrast, in a different instance `The data suggests that *profilin* binding to actin weakens nucleotide binding to actin by disrupting Mg(2+) coordination in the actin central cleft.’ the key terms `*profilin*’ and `*actin*’ are correlated as direct-interacting partners. Since the contextual nature for the association is distinct from the previous ones, the linguistic representation [RELATION IN PROTEINAGENT ➔ PROTEINAGENT NN TO ➔ PROTEINTARGET IN VBN] also differs. In essence, the method efficiently captures the variations in sentential inferences and reproduces them in corresponding linguistic patterns. It should be noted that all parts of the contextual patterns are used in refining the tree structure for the classifier kernel.

## Concluding remarks

Our paper introduces a modified tree kernel-based representation for identifying different bio-entity relations from PubMed abstracts like chemical–protein, chemical-induced disease and PPI. The complex multi-class intra-sentence interactions and the class-size bias in selective corpora made sematic relation detection especially challenging. We attempted to establish a model, which resourced feature optimization to enhance the tree kernel performance. The method yields better results in binary classification, while its recall remains competent with other methods irrespective of the size of the classification labels. One of the promising aspects of this method is its ability to generate exhaustive linguistic patterns fashioned from invariant features. These patterns are readable, explicit and reusable in tasks with similar contexts. Therefore, we conjecture that our method can be highly effective as a feature generation tool, which can be applied to diverse scenarios. Besides introducing the linguistic patterns, the proposed method also explores a novel way of text representation by projecting text as conic section. This opens up a new domain requiring additional experimentation with other features of conic representation that can be potentially used with different classifiers. Geometric figures represent a more coherent form, which makes linguistic variations and sentential transformations easier to understand, identify and evaluate. Despite our best efforts, the performance of our system in multi-class scenario stagnated. Hence, in alignment with our error analysis, we plan to refurbish our optimization module with additional features based on selective KBs and deep learning-based strategies in the future. Moreover, we also encountered some issues related to cross-class categorization owing to the similarity in representational patterns. As a result, to strengthen our algorithm, we plan to experiment with dynamic contexts in invariance identification and explore alternative invariant representations within text for improved classification.

## Supplementary Material

Supplementary Data
